# Atrial fibrillatory rate as predictor of recurrence of atrial fibrillation in horses treated medically or with electrical cardioversion

**DOI:** 10.1111/evj.13551

**Published:** 2022-01-13

**Authors:** Rikke Buhl, Eva M. Hesselkilde, Helena Carstensen, Charlotte Hopster‐Iversen, Gunther van Loon, Annelies Decloedt, Glenn Van Steenkiste, Celia M. Marr, Virginia B. Reef, Colin C. Schwarzwald, Katharyn J. Mitchell, Katarina Nostell, Nora Nogradi, Søren S. Nielsen, Jonas Carlson, Pyotr G. Platonov

**Affiliations:** ^1^ Department of Veterinary Clinical Sciences Faculty of Health and Medical Sciences University of Copenhagen Copenhagen Denmark; ^2^ Equine Cardioteam Department of Large Animal Internal Medicine Ghent University Merelbeke Belgium; ^3^ Rossdales Equine Hospital Newmarket UK; ^4^ Department of Clinical Studies New Bolton Center University of Pennsylvania School of Veterinary Medicine Kennett Square Pennsylvania USA; ^5^ Equine Department Vetsuisse Faculty University of Zurich Zurich Switzerland; ^6^ Department of Clinical Sciences Faculty of Veteirnary Sciences Swedish University of Agricultural Sciences Uppsala Sweden; ^7^ Dubai Equine Hospital Dubai UAE; ^8^ Department of Veterinary Sciences Faculty of Health and Medical Sciences University of Copenhagen Copenhagen Denmark; ^9^ Department of Cardiology Lund University Lund Sweden

**Keywords:** AFR, arrhythmia, cardiology, ECG, electrophysiology, horse, surface electrocardiogram

## Abstract

**Background:**

The recurrence rate of atrial fibrillation (AF) in horses after cardioversion to sinus rhythm (SR) is relatively high. Atrial fibrillatory rate (AFR) derived from surface ECG is considered a biomarker for electrical remodelling and could potentially be used for the prediction of successful AF cardioversion and AF recurrence.

**Objectives:**

Evaluate if AFR was associated with successful treatment and could predict AF recurrence in horses.

**Study design:**

Retrospective multicentre study.

**Methods:**

Electrocardiograms (ECG) from horses with persistent AF admitted for cardioversion with either medical treatment (quinidine) or transvenous electrical cardioversion (TVEC) were included. Bipolar surface ECG recordings were analysed by spatiotemporal cancellation of QRST complexes and calculation of AFR from the remaining atrial signal. Kaplan‐Meier survival curve and Cox regression analyses were performed to assess the relationship between AFR and the risk of AF recurrence.

**Results:**

Of the 195 horses included, 74 received quinidine treatment and 121 were treated with TVEC. Ten horses did not cardiovert to SR after quinidine treatment and AFR was higher in these, compared with the horses that successfully cardioverted to SR (median [interquartile range]), (383 [367‐422] vs 351 [332‐389] fibrillations per minute (fpm), *P* < .01). Within the first 180 days following AF cardioversion, 12% of the quinidine and 34% of TVEC horses had AF recurrence. For the horses successfully cardioverted with TVEC, AFR above 380 fpm was significantly associated with AF recurrence (hazard ratio = 2.4, 95% confidence interval 1.2‐4.8, *P* = .01).

**Main limitations:**

The treatment groups were different and not randomly allocated, therefore the two treatments cannot be compared. Medical records and the follow‐up strategy varied between the centres.

**Conclusions:**

High AFR is associated with failure of quinidine cardioversion and AF recurrence after successful TVEC. As a noninvasive marker that can be retrieved from surface ECG, AFR can be clinically useful in predicting the probability of responding to quinidine treatment as well as maintaining SR after electrical cardioversion.

## 1 INTRODUCTION

Atrial fibrillation (AF) is the most common sustained cardiac arrhythmia in horses with an estimated prevalence of 0.1%‐2.5% depending on breed and the sample studied.^1^, ^2^, ^3^, ^4^ Atrial fibrillation is associated with substantially reduced athletic performance^5^, ^6^ and is therefore a clinically important disease. Treatment options include medical treatment and electrical cardioversion. The most used medical treatment is nasogastric administration of quinidine sulphate that successfully restores sinus rhythm (SR) in 80% of the reported cases. The development of severe adverse effects such as colic, and ventricular tachyarrhythmias may necessitate cessation of the treatment before cardioversion has occurred.^7^ Other medical treatments have been applied to cases with both induced and spontaneous AF, however, all with lower success rates.^8^, ^9^, ^10^, ^11^, ^12^ Transvenous electrical cardioversion (TVEC) has a high success rate, where 96% of the cases reported are successfully cardioverted.^7^ The high cardioversion rates are, however, followed by high AF recurrence rates (15%‐43%) regardless of treatment method.^13^, ^14^, ^15^, ^16^ Atrial fibrillation is a complex disease that remodels the atria, which further promotes AF and the high recurrence rates are believed to be linked to this remodelling.^17^, ^18^ Although factors such as breed, AF duration, number of previous AF episodes, valvular regurgitation, atrial size and contractile function as well as increased numbers of atrial premature beats following cardioversion are reported as negative prognostic indicators in horses,^13^, ^15^, ^16^ additional biomarkers that are objectively quantified indicating the degree of remodelling and therefore, potential AF recurrence, are warranted. An acknowledged hallmark of atrial remodelling is shortening of the atrial effective refractory period (aERP)^19^ and thus shortening of atrial fibrillatory cycle length (AFCL). The mean AFCL can be measured invasively with a catheter placed in the atrium and has been extensively studied in the right atrium of horses with AF.^9^, ^20^, ^21^ The atrial fibrillatory rate (AFR) measures the number of fibrillations (f waves) per minute (fpm) and is inversely correlated with AFCL. The AFR can be measured noninvasively from surface ECG recordings and has received considerable attention in horses over the recent years.^12^, ^22^, ^23^, ^24^, ^25^ Both the AFCL and the AFR are generally accepted as surrogate markers for atrial electrical remodelling.^26^ Studies in horses have shown the correlation between AF duration and AFR or AFCL, indicating electrical remodelling over time.^20^, ^23^, ^27^ Correlation between AFCL measured invasively and AFR derived from the surface ECG has recently been investigated.^25^ Also, changes in AFR in response to treatment with antiarrhythmic drugs and towards cardioversion, both with and without drug interventions, have been reported in horses.^22^, ^24^ In human AF patients, AFR and AFCL have been proposed as prognostic biomarkers for monitoring the effect of antiarrhythmic drugs^28^, ^29^, ^30^, ^31^ and baseline AFR has been proposed as a predictor of spontaneous cardioversion.^32^

The objective of this study was to evaluate if AFR derived from surface ECG could predict the outcome of AF treatment in horses. The aim of this study was therefore (1) to evaluate if AFR was associated with successful cardioversion and (2) to evaluate if AFR was associated with AF recurrence after successful treatment. We hypothesised that (1) AFR was higher in horses that did not cardiovert to SR after treatment and (2) horses with high AFR had a higher recurrence rate.

## 2 MATERIALS AND METHODS

Seven referral centres contributed with clinical records and ECG recordings of horses presented with AF for longer than 48 hours from January 2008 to November 2019 (Ghent University [85 horses], University of Pennsylvania [39 horses], Rossdales Equine Hospital [27 horses], University of Copenhagen [19 horses], University of Zurich [17 horses], Swedish University of Agricultural Sciences [17 horses] and Dubai Equine Hospital [nine horses]).

The inclusion criteria were as follows: (1) admission for cardioversion using quinidine or TVEC with persistent AF, (2) availability of ECG with AF exported in digital format and (3) ECG recorded at rest prior to any treatment. Also, cardiac auscultation with murmurs graded on a 1‐6/6 scale and echocardiographic examination at rest before cardioversion should preferably be available. From the study period, AF episodes were collected from 213 horses. Each horse was only included once in the data set and only for one treatment (either quinidine or TVEC).

**FIGURE 1:**
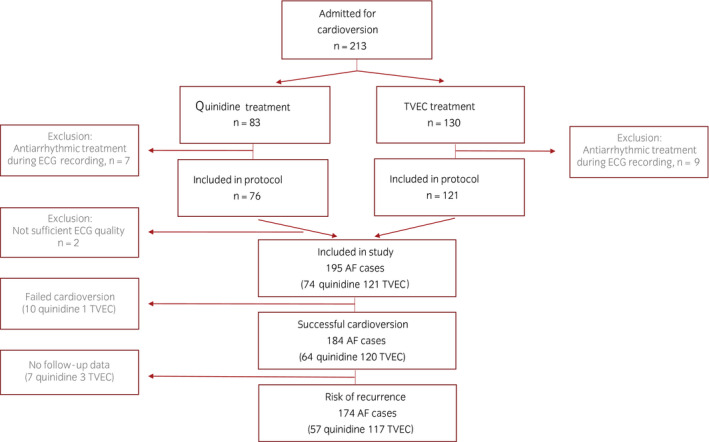
Schematic flowchart of the horses included in the study and reasons for exclusion of horses

Of the 213 horses, ECGs from 18 horses were excluded due to either a poor‐quality recording or that ECG recordings were only available during antiarrhythmic treatment, leaving 195 horses to be included in the study (Figure 1).

Echocardiographic measurements included maximum left atrial area (LAA) and left atrial diameter measured at end ventricular systole prior to mitral opening from the right parasternal four‐chamber view as well as left ventricular diameter measured at end‐diastole from a right parasternal short‐axis view at the chordal level. Also, the classification of valvular regurgitation was registered. Mitral and tricuspid regurgitation were classified by area of the regurgitation from Colour Flow Doppler occupying the receiving atrium area and classified in the following categories: none, trivial, mild, moderate and severe. Aortic regurgitation was classified by the diameter of the regurgitation compared with left ventricular outflow tract from Colour Flow Doppler and classified in the following categories: none, trivial, mild, moderate and severe. The measurements were provided by each referral centre and therefore conducted by several observers. The AF duration was estimated based on the clinical records and anamneses, where the referring veterinarian, owner, trainer or rider estimated AF duration. Previous AF treatment was registered and the duration of the follow‐up period as well as eventual AF recurrence within 180 days after treatment were based on information from the medical record.

The following information from the treatment procedures was registered: treatment with quinidine or TVEC. For quinidine treatment (via nasogastric intubation), dose per treatment (mg/kg), number of doses given and total quinidine dose (g) for the treatment period were recorded. For TVEC treatment, the cardioverting energy (J), total energy delivery (J) and the number of shocks were recorded. For all horses, it was noted if additional antiarrhythmic drugs were given before, during or after treatment. Treatment outcome (cardioversion to SR or not) was registered. Modified base‐apex ECG recordings were exported as raw data files for off‐line processing. Three 1‐minute intervals from the ECG recordings were used for the analyses.^24^ If more than three 1‐minute ECG recordings were analysed, the first three 1‐minute recordings, where >80% of the atrial signals was used for the analyses, were selected. The 1‐minute recordings followed each other consecutively with no time span in between. If less than three 1‐minute ECG recordings were of acceptable quality for analysis, one 1‐minute ECG recording was included. Analyses and selection of the intervals were performed by one operator without any knowledge of clinical data. The ECG analyses, including QRST cancellation and AFR calculation, were performed using the AFR software (Cardiolund AFR Tracker, Cardiolund) as previously described.^33^, ^34^ In short, ECG signals were preprocessed (digital ECG data from different sources adapted to the input format required by the analysis software), filtered, and QRST signals removed by spatiotemporal cancellation. The remaining residual ECG then mainly contained atrial activity and from these, the AFR could be calculated over a specified time period, which is reported as the AFR expressed in fibrillations per minute.

Electrocardiograms from three horses had been used in a previous study,^22^ ECGs from another 73 horses have been included in a recent study^25^ and 19 of those have also been used in a second study.^20^

### 2.1 Data analysis

Descriptive analysis was used to compare demographics based on age, sex, body weight, breed and use. Categorical variables were expressed as a percentage and continuous variables were presented either by means and standard deviations (SD) for normally distributed data or as median (interquartile range, IQR) for nonparametric data. Normality was checked by visual inspection of the raw data plots and by using the Shapiro‐Wilk test of linearity.

Friedman's test for comparison of paired groups was used for assessment of AFR stability by testing differences between AFR values calculated from the three 1‐minute recordings.

Differences between the horses that were cardioverted with quinidine and by TVEC were examined using Chi‐square tests for categorical variables, independent *t* tests for continuous, normally distributed variables and Mann‐Whitney *U* test for data that were not normally distributed.

For the horses treated with quinidine, differences of AFR between successful and no successful cardioversion was examined using Mann‐Whitney *U* test. Additionally, the value of AFR for prediction of SR restoration following quinidine administration was assessed using ROC curve analysis. As only one horse did not cardiovert to SR with TVEC, these analyses could not be conducted for the TVEC group.

Risk assessment of AF recurrence by 180 days after cardioversion was studied using Kaplan‐Meier survival curve and Cox regression analyses in those animals who underwent successful cardioversion and were included in the longitudinal follow‐up. A log‐rank test was used to compare survival probabilities between the groups. Proportional hazard assumption was tested using Schoenfeld partial residuals method. The horses were divided into groups based on the intervention (quinidine or TVEC) and the analyses were performed separately for the quinidine and TVEC treated animals. The association between AFR and the likelihood of AF recurrence after cardioversion was assessed using Kaplan‐Meier survival curve and Cox regression analyses. Horses were censored at the time of the last follow‐up if no AF recurrence had occurred until then. For the Kaplan‐Meier analysis, AFR was dichotomised by median values separately for the quinidine (median value 350 fpm) and TVEC (median value 376 fpm, but for simplicity, we chose 380 fpm as the close to median value) treated animals and the significance of differences between the groups depending on the AFR value was assessed using log‐rank *P* value. Cox regression analysis for assessment of the relationship between AFR and the risk of AF recurrence was performed using AFR as a continuous variable and dichotomised by the close to median value as explained above. The results of Cox regression analysis are presented as unadjusted hazard ratio (HR) as well as HR adjusted for clinically relevant covariates (age, breed, duration of AF prior to cardioversion, moderate or severe mitral regurgitation measured by echocardiography, LAA and administration of antiarrhythmic drugs following restoration of SR).

The significance threshold was set at *P* < .05. Statistical analyses were performed using SPSS version 26.0 and GraphPad Prism, Version 8.4.3.

## 3 RESULTS

### 3.1 Case selection and demographical characteristics

Of the 195 horses included, 167 had no history of previous AF, whereas 28 horses had been treated for AF before and now presented with recurrence. Seventy‐four horses were treated with quinidine (two horses received quinidine gluconate intravenously and the specific dose for these two horses were not registered, whereas the remaining horses received 22 mg/kg quinidine sulphate via nasogastric intubation administered with an increasing number of doses over one or more days). Of the 74 horses receiving quinidine, 21 were treated at Rossdales Equine Hospital, 16 at Swedish University of Agricultural Sciences, 16 at University of Copenhagen, 10 at University of Pennsylvania, 9 at University of Zurich and 2 at Dubai Equine Hospital. One hundred and twenty‐one horses were treated with TVEC (85 Ghent University, 15 University of Pennsylvania, eight University of Zurich, seven Dubai Equine Hospital, five Rossdales Equine Hospital, and one at University of Copenhagen). Increasing energy delivery (J) was applied for each shock until cardioversion occurred. Eleven horses did not cardiovert to SR (10 quinidine and 1 TVEC treated horse) and another 10 horses had no follow‐up data on AF recurrence. Therefore, in total, 174 horses were subjected to the Kaplan‐Meier and Cox regression analyses to investigate the time to AF recurrence (flowchart, see Figure 1). Clinical characteristics for horses successfully cardioverted by quinidine or TVEC including breed, body weight, age, cardiac murmurs, valvular regurgitations, left atrial and ventricular size including the number of animals where the variable had been registered are summarised in Table 1. The majority of horses included in the TVEC group were Warmbloods (84%), whereas racehorses accounted for 58% of the horses included in the quinidine group. Horses treated with TVEC were significantly heavier, older, had a higher prevalence of mitral regurgitation and larger LAA, compared with the horses treated with quinidine (Table 1).

**TABLE 1:** Clinical characteristics for horses successfully cardioverted on quinidine (left) and with transvenous electrical cardioversion (TVEC; right). Categorical variables were expressed as a percentage and continuous variables were presented either by means and standard deviations (SD) for normally distributed data or as median (interquartile range, IQR) for nonparametric data. The *P* value indicates that there is a statistically significant difference when the quinidine and TVEC groups are compared in univariable analysis, and n is the number of animals where the variable has been registered in the clinical records

Variables	Successful quinidine cardioversion n = 64	n	Successful TVEC cardioversion n = 120	n	*P* value
Breed (%)	31% Warmblood 25% Standardbred 33% Thoroughbred 11% other breeds	64	84% Warmblood 10% Standardbred 3% Thoroughbred 3% other breeds	120	<0.0001
Use (%)	13% showjumping 5% dressage 52% racing 15% eventing/endurance 15% pleasure	62	43% showjumping 23% dressage 13% racing 10% eventing/endurance 11% pleasure	119	<0.0001
Sex (%)	5% stallion 64% gelding 31% mare	64	12% stallion 60% gelding 28% mare	120	0.4
Body weight (kg)	544 ± 77	64	583 ± 69	118	<0.001
Age (y)	6 [4‐9]	64	9 [7‐12]	120	<0.0001
Systolic murmur left side (%)	89% No murmur 11% grade 1‐3 0% grade 4‐6	56	72% No murmur 21% grade 1‐3 7% grade 4‐6	118	<0.001
Diastolic murmur left side (%)	100% No murmur 0% grade 1‐3 0% grade 4‐6	56	92% No murmur 8% grade 1‐3 0% grade 4‐6	119	0.08
Systolic murmur right side (%)	82% No murmur 18% grade 1‐3 0% grade 4‐6	57	81% No murmur 17% grade 1‐3 2% grade 4‐6	118	0.9
Mitral regurgitation (%)	79% None/Trivial 18% Mild 3% Moderate/Severe	56	54% None/Trivial 37% Mild 9% Moderate/Severe	119	0.01
Tricuspid regurgitation (%)	66% None/Trivial 29% Mild 5% Moderate/Severe	56	64% None/Trivial 26% Mild 10% Moderate/Severe	119	0.6
Aortic regurgitation (%)	88% None/Trivial 12% Mild 0% Moderate/Severe	56	73% None/Trivial 21% Mild 6% Moderate/Severe	119	0.07
LAD (cm)	12.6 ± 1.2	53	12.9 ± 1.3	112	0.5
LAA (cm^2^)	98 ± 15	39	106 ± 21	116	0.048
LVID (cm)	11.8 ± 1.2	55	11.8 ± 1.0	115	0.7

Abbreviations: LAA, left atrial diameter; LAD, left atrial diameter; LVID, left ventricular internal diameter.

Of the 184 horses successfully cardioverted to SR, 17 had only one 1‐minute ECG recording of acceptable quality for AFR analysis. For the remaining horses, no difference was found between the three 1‐minute recordings (mean ± SD values; AFR minute 1:368 ± 46 fpm, AFR minute 2:367 ± 46 fpm, AFR minute 3:367 ± 47 fpm, *P* = .4) and therefore only the first 1‐minute AFR recording was used for the following analyses.

The AF characteristics, such as the estimated AF duration before treatment, recurrence rate, AFR, as well as treatment information, are summarised in Table 2. Duration of AF was longer for the successfully cardioverted horses in the TVEC group compared with the quinidine group. The recurrence rate 180 days after cardioversion and the AFR before treatment were lower in the quinidine group compared with horses treated with TVEC (Table 2). For the successfully cardioverted horses receiving quinidine treatment the median number of treatments (dosage 22 mg/kg) was three and 18 horses (28%) received additional antiarrhythmic drugs during quinidine treatment. No horses in this group were treated with antiarrhythmic drugs following cardioversion to SR (Table 2). For the successfully cardioverted horses in the TVEC group, the median number of shocks required for cardioversion was two and a relatively large proportion of horses received antiarrhythmic drugs before (45 [28%] horses), during (26 [22%] horses) and after (53 [45%] horses) TVEC treatment (Table 2). The AFR at cardioversion attempt regardless of the outcome, also including the horses that were not successfully cardioverted, was 357 (IQR 335‐394] fpm in the quinidine group and 376 (IQR 350‐402) fpm in the TVEC group (*P* = .03). The ten noncardioverting horses treated with quinidine consisted of seven Warmbloods, one Standardbred and two other breeds, eight were geldings and two mares. Body weight was 624 ± 67 kg, age 9 (8‐13) years, three horses had systolic murmur on the left side (grade 2‐3/6) and six of the horses had mild or moderate mitral regurgitation. They had AF for 120 (45‐195) days before the cardioversion attempt.

**TABLE 2:** Atrial fibrillation (AF) duration, recurrence rate, follow‐up time, atrial fibrillation rate (AFR) and treatment characteristics for horses successfully cardioverted on quinidine and with transvenous electrocardioversion (TVEC). Categorical variables were expressed as a percentage and continuous variables were presented as median (interquartile range, IQR) for nonparametric data. The *P* value indicates that there is a statistically significant difference when the quinidine and TVEC groups are compared in univariable analysis, and n is the number of animals where the variable has been registered in the clinical records

Variables	Successful quinidine cardioversion n = 64	N	Successful TVEC cardioversion n = 120	n	*P*‐value
AF duration (d)	16 [7‐38]	48	45 [28‐120]	110	<0.0001
Recurrence <180 d (%)	12% Yes 88% No	57	34% Yes 66% No	117	0.002
Follow‐up time (d)	365 [167‐755]	57	365 [150‐700]	117	0.6
AFR (fpm)	351 [332‐389]	64	376 [350‐403]	120	0.003
Number of doses (22 mg/kg)	3 [2‐4]	62			
Total dose (g) before cardioversion	36 [22‐51]	61			
Antiarrhythmics^a^ added to quinidine treatment	28%	53			
Numbers of shocks			2 [1‐4]	107	
Total energy (J)			350 [150‐876]	106	
Cardioverting energy (J)			200 [150‐250]	107	
Antiarrhythmics^b^ before TVEC			38%	119	
Antiarrhythmics^b^ during TVEC			22%	118	
Antiarrhythmics^b^ after TVEC			45%	119	

^a^ Antiarrhythmic drugs added during quinidine treatment registered: Digoxin, ACE inhibitors, flecainide.

^b^ Antiarrhythmic drugs added before, during and after TVEC registered: Sotalol, amiodarone, digoxin or a combination of antiarrhythmic drugs.

### 3.2 Immediate cardioversion success

Sinus rhythm was restored in 64 (86%) of the horses receiving quinidine, while in 10 horses, cardioversion was unsuccessful. The AFR assessed amongst noncardioverters from the quinidine group was significantly higher than in those who cardioverted successfully (383 [IQR 367‐422] vs 351 [IQR 332‐389] fpm, *P* < .01). The ROC analyses showed that AFR was significantly associated with the likelihood of AF cardioversion to SR (c‐statistics 0.770).

In the TVEC group, SR was restored in all (99%) but one horse (AFR 402 fpm) and therefore no statistical analysis was deemed applicable.

### 3.3 AF recurrence during follow‐up

#### 3.3.1 Quinidine group

**FIGURE 2:**
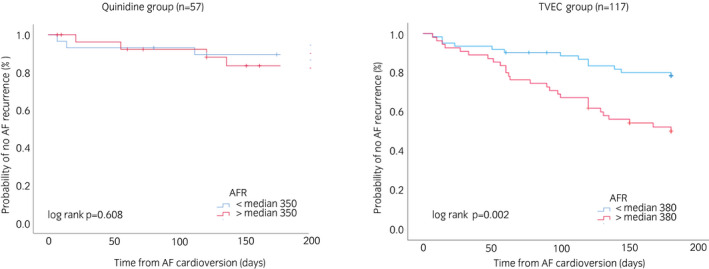
Kaplan‐Meier survival curves with log‐rank *P* values for quinidine and transvenous electrocardioversion (TVEC) group for the probability of atrial fibrillation (AF)‐free survival, stratified by the atrial fibrillatory rate (AFR) (median AFR 350 fpm for quinidine treatment and 380 fpm for TVEC treatment) on the day of admission for cardioversion

In total, 57 horses were included in the follow‐up after the successful restoration of SR by quinidine. Median AFR at cardioversion in the follow‐up cohort was 350 (IQR 328‐378). No difference in the median AFR values was observed between the 7 horses (12%) who experienced AF recurrence within the first 180 days (352 [IQR 339‐365]) and 50 horses (88%) who did not (350 [IQR 325‐381]; *P* = .87). In the Kaplan‐Meier curve analysis using AFR value dichotomised by median, no difference was observed between the groups with regard to the likelihood of AF recurrence during the follow up (log‐rank *P* = .608, Figure 2).

#### 3.3.2 TVEC group

A total of 117 horses were included in the follow‐up after the successful restoration of SR by TVEC. Median AFR before cardioversion in the follow‐up cohort was 376 [IQR 351‐403]. Within the first 180 days, 40 (34%) of the horses treated with TVEC had AF recurrence (402 [IQR 376‐422]) and 77 (66%) did not (365 [IQR 340‐392]; *P* < .001). The AFR was significantly associated with the risk of recurrence in the Kaplan‐Meier survival curve and Cox regression analyses. The Kaplan‐Meier analysis (Figure 2) demonstrates that elevated AFR was associated with the risk of AF recurrence. In the Cox regression analysis, increased AFR was significantly associated with AF recurrence both in the univariable analysis and after adjustment for clinically relevant covariates (Table 3).

**TABLE 3:** Cox regression analysis of the transvenous electrocardiversion (TVEC) group with continuous atrial fibrillation rate (AFR) and AFR dichotomised by 380 fpm. The hazard ratio (HR) for atrial fibrillation (AF) recurrence at 180 d is shown for the univariable model and for the adjusted model considering clinically relevant covariates (age, breed, duration of AF, moderate or severe mitral regurgitation, left atrial area and the use of antiarrhythmic drugs after cardioversion)

	Univariable model			Adjusted model		
	HR	95% CI	*P* value	HR	95% CI	*P* value
TVEC group (n = 117)						
AFR continuous	1.017	1.009‐1.026	<0.01	1.012	1.004‐1.020	0.004
AFR > 380 vs AFR ≤ 380	2.771	1.428‐5.379	0.003	2.424	1.217‐4.827	0.01

Abbreviations: CI, confidence interval; HR, hazard ratio.

## 4 DISCUSSION

This study is the first multicentre study to address electrical remodelling measured by AFR as a predictor of cardioversion success and AF recurrence in horses. We found that higher AFR was associated with the failure of quinidine cardioversion and that higher AFR was associated with a higher risk of AF recurrence for horses cardioverted with TVEC. Amongst horses that responded to quinidine treatment, AF recurrence rate during follow‐up was low and not associated with AFR.

Atrial fibrillation results in structural, electrical and functional remodelling of the atria, and delayed or insufficient reversibility of these changes will lead to increased inducibility and sustainability of the disease, which may prevent maintenance of SR after cardioversion.^35^

Atrial fibrillatory rate is a measure of electrical remodelling and has recently been applied in equine AF research for studying disease development over time in both horses with induced AF^12^, ^23^ and horses with spontaneous persistent AF.^22^, ^25^ In addition, the effect of antiarrhythmic treatment on the AFR^12^, ^22^, ^24^ has been studied. These studies have shown that AFR increases in experimentally induced acute AF, where the AFR is reported to be approximately 300 fpm,^12^, ^22^ increasing to approximately 350 fpm after 30 days of AF^12^ and to 376 fpm after 2 months of AF.^23^ In human studies, it has been shown that low‐frequency fibrillation was more probably to terminate spontaneously or respond to antiarrhythmic therapy, whereas high‐frequency fibrillation was more persistent and drug refractory^30^, ^36^, ^37^ and therefore AFR has been suggested as a predictor of cardioversion success. Clinical studies in human patients have reported AFR cut‐off values predicting spontaneous cardioversion being <355 fpm.^26^, ^32^ In horses with induced AF, it was also shown that low AFR is more probably to terminate spontaneously compared with horses with high AFR values.^22^ This is similar to the findings of the current study where horses with unsuccessful quinidine cardioversion had higher AFR compared with horses where cardioversion was successful. As almost all horses cardioverted due to the very high success rate of TVEC, we were not able to calculate the predictive value of AFR for TVEC.

In the current study, we found a significant difference in AFR between horses relapsing into AF and those maintaining SR over a 180‐day follow‐up period for the horses treated with TVEC. The findings correspond to studies in human AF patients, where AFR was higher in patients with AF recurrence the following cardioversion.^30^, ^36^, ^37^, ^38^ This has also been observed in a study including 18 horses electrically cardioverted, where the shortest fifth percentile of AFCL (corresponding to high AFR) has been reported as a risk factor of AF recurrence.^20^ The above‐mentioned findings all indicate that remodelling, whether it is electrical or structural, resulting in higher AFR will increase the risk of AF recurrence after cardioversion. Based on the clinical characteristics, there are noticeable differences between the horses successfully treated with quinidine and with TVEC, and therefore, the outcome of the two groups cannot be directly compared. The TVEC group included larger and older horses (primary Warmbloods), which had a higher prevalence of mitral regurgitation, larger LAA and longer duration of AF. Similar phenotypic characteristics were observed for the ten noncardioverting horses treated with quinidine. The described clinical characteristics of the noncardioverting quinidine treated horses and the TVEC group are in concurrence with risk factors for AF previously reported such as mitral regurgitation, increased LAA and AF duration.^13^, ^15^, ^39^ The combination of high AFR and increased LAA has predicted early AF recurrence after successful cardioversion in human patients, providing individual risk estimates in human AF patients.^28^ A certain size of the atria is believed to maintain a critical high number of re‐entry circuits and large atria therefore can accommodate a higher number of co‐existing f waves resulting in increased AFR.^40^ The findings in the current study suggest more remodelled atria in the TVEC group and therefore, this group is most probably more prone to AF recurrence compared with the quinidine group.

In human patients, the AF duration affects the significance of AFR in predicting AF recurrence. For AF episodes less than 30 days duration there was a high correlation between AFR and AF recurrence rate, although no correlation could be found for episodes lasting longer than the 30 days.^38^ Previous studies in horses have shown that AF duration less than four months had a lower recurrence of AF compared with the horses with longer AF duration before treatment attempt.^13^, ^39^ In the current study, the predictive value of AFR for AF recurrence remained significant after adjustment for the duration of AF. However, the duration of AF is not precise, as it was estimated based on the time of diagnosis by the veterinarian, owner or trainer. Therefore, AF duration may be underestimated especially in horses not performing intensive physical exercise.

We found that the AF recurrence rate within the first 180 days after cardioversion was significantly lower for the quinidine‐treated horses (12%), compared with the TVEC‐treated horses (34%). This most probably reflects differences in study samples with regards to factors predisposing for AF recurrence (eg duration of AF, breed, body weight, age, mitral regurgitation) and was influenced by the referral centres' treatment preference and caseload, and therefore, the two groups should not be directly compared. As the follow‐up period varied between horses, with some horses being followed for many years, whereas others only for weeks or months, and to reduce the number of dropouts over time, we only reported recurrence rate within the first 180 days after cardioversion. Therefore, we cannot directly compare our recurrence rates with the results of previous studies as they mostly have longer (or not specified) follow‐up periods. Consequently, we report lower recurrence rates but with similar breed differences, where studies including main racehorses report lower recurrence rates (15%‐30% recurrence),^13^, ^14^ compared with studies dominated by Warmbloods (33%‐43% recurrence).^15^, ^16^

In a multicentre study with retrospective data collection, there is a risk for centre‐dependent differences in management strategies not reflected in the selected clinical‐relevant covariates used in the adjusted model. However, the inclusion of the site in the adjusted model did not affect the results and yielded similar HR for the association between AFR and AF recurrence after cardioversion (HR 2.310, 95% CI 1.109‐4.813, *P* = .025 for AFR dichotomised by median).

Two horses in the quinidine group were treated with quinidine gluconate intravenously. Both quinidine gluconate and quinidine sulphate are salts of quinidine, which blocks the sodium channels and are categorised as class Ia antiarrhythmic drugs. The absorption of quinidine gluconate via nasogastric administration is lower than quinidine sulphate^41^ and is therefore administered intravenously. As the active pharmacological compound of both quinidine gluconate and quinidine sulphate are similar, we decided to include the two horses in the group.

As we did not include ECGs, where the horses were treated with any antiarrhythmic drugs at the time of recording, the general effect of drugs on AFR could not be assessed in this study. However, studies in both human patients and horses have clearly shown that AFR decreases in response to medical treatment and therefore AFR might aid the clinician in following the effect of medical antiarrhythmic treatment.^22^, ^24^, ^29^ Also, early patient selection may be especially valuable since pharmacological cardioversion has a lower success rate than electrical cardioversion and therefore, a horse with high AFR may potentially have a higher chance of cardioverting by TVEC rather than quinidine. At the current time, the ECG analysis is post‐processing, but future ECG recording and analysis systems may be able to calculate AFR instantaneously, which would provide the clinician with information that can assist not only in treatment selection but also in prognostication of the risk of AF recurrence.

### 4.1 Limitations

Due to the retrospective nature, the data set is based on medical records, and the follow‐up strategy varies between the centres where some active follow‐up the patients, whereas others have no follow‐up strategies. Therefore, the detailed treatment as well as the follow‐up protocols may not have been identical between the centres. Duration of AF is difficult to estimate, especially for riding or pleasure horses where AF may have been present for weeks or months unnoticed by the owner or rider. In contrast, AF will seldom be ignored in racehorses due to its performance‐limiting effect. These horses are therefore often treated early when the atria are less remodelled and fewer AF recurrences are seen. Finally, the two treatment groups were of variable size, with fewer quinidine cases, and the horses were not randomly allocated to the groups. The low number of horses treated with quinidine affects the strength of the findings in this subgroup and therefore, the findings related to the use of quinidine should be interpreted with caution.

## 5 CONCLUSION

The current study applied a signal‐processing technique, which noninvasively measured the dominant frequency of AF waves from a bipolar surface ECG as a biomarker of electrical remodelling. We included 195 horses from seven different referrals centres making this the largest known AF study in horses. We demonstrated that AFR was associated with cardioversion success following quinidine treatment, and for horses treated with TVEC, higher AFR was associated with an increased risk of AF recurrence. Useful indices to identify candidates with a high risk of AF recurrence after cardioversion are needed, and the AFR could be clinically valuable for predicting the outcome of maintaining SR after cardioversion.

## CONFLICT OF INTERESTS

No competing interests have been declared.

## AUTHOR CONTRIBUTIONS

R. Buhl and E. Hesselkilde participated in study design, study execution, data analyses and interpretation and preparation of the manuscript. R. Buhl also has full access to the data and takes responsibility for the integrity and accuracy of data. H. Carstensen participated in the study design and preparation of the manuscript. C. Hopster‐Iversen, G. van Loon, A. Decloedt, G. Van Steenkiste, C. Marr, V. Reef, C. Schwarzwald, K. Mitchell, K. Nostell, N. Nogradi, J. Carlson and P. Platonov participated in study execution, and data analyses and interpretation. S. Nielsen participated in study execution, and data analyses and interpretation. All authors gave their final approval of the manuscript.

## ETHICAL ANIMAL RESEARCH

The local ethical committee at the Department of Veterinary Clinical Sciences, University of Copenhagen approved the study.

## INFORMED CONSENT

Owner informed consent is not stated for all hospitals involved.

### PEER REVIEW

The peer review history for this article is available at https://publons.com/publon/10.1111/evj.13551.

## Data Availability

The data are available from the corresponding author on reasonable request.
